# Crystal Chemistry of the Copper Oxalate Biomineral Moolooite: The First Single-Crystal X-ray Diffraction Studies and Thermal Behavior

**DOI:** 10.3390/ijms24076786

**Published:** 2023-04-05

**Authors:** Ilya V. Kornyakov, Vladislav V. Gurzhiy, Mariya A. Kuz’mina, Maria G. Krzhizhanovskaya, Nikita V. Chukanov, Mikhail V. Chislov, Anatolii V. Korneev, Alina R. Izatulina

**Affiliations:** 1 Department of Crystallography, Institute of Earth Sciences, St. Petersburg State University, University Emb. 7/9, 199034 Saint-Petersburg, Russia; ikornyakov@mail.ru (I.V.K.); m.kuzmina@spbu.ru (M.A.K.); krzhizhanovskaya@mail.ru (M.G.K.); a_v_korneev@list.ru (A.V.K.); 2 Laboratory of Nature-Inspired Technologies and Environmental Safety of the Arctic, Kola Science Centre, Russian Academy of Sciences, Fersmana 14, 184209 Apatity, Russia; 3 Institute of Problems of Chemical Physics, Russian Academy of Sciences, 142432 Chernogolovka, Russia; chukanov@icp.ac.ru; 4 Center of Thermal Analysis and Calorimetry, St. Petersburg State University, University Emb. 7/9, 199034 Saint-Petersburg, Russia; mihailchislov@gmail.com

**Keywords:** copper oxalate, moolooite, bioorganic nanostructure, biomineralogy, X-ray diffraction, diffuse scattering, structural disorder

## Abstract

Moolooite, Cu(C_2_O_4_)·*n*H_2_O, is a typical biomineral which forms due to Cu-bearing minerals coming into contact with oxalic acid sources such as bird guano deposits or lichens, and no single crystals of moolooite of either natural or synthetic origin have been found yet. This paper reports, for the first time, on the preparation of single crystals of a synthetic analog of the copper-oxalate biomineral moolooite, and on the refinement of its crystal structure from the single-crystal X-ray diffraction (SCXRD) data. Along with the structural model, the SCXRD experiment showed the significant contribution of diffuse scattering to the overall diffraction data, which comes from the nanostructural disorder caused by stacking faults of Cu oxalate chains as they lengthen. This type of disorder should result in the chains breaking, at which point the H_2_O molecules may be arranged. The amount of water in the studied samples did not exceed 0.15 H_2_O molecules per formula unit. Apparently, the mechanism of incorporation of H_2_O molecules governs the absence of good-quality single crystals in nature and a lack of them in synthetic experiments: the more H_2_O content in the structure, the stronger the disorder will be. A description of the crystal structure indicates that the ideal structure of the Cu oxalate biomineral moolooite should not contain H_2_O molecules and should be described by the Cu(C_2_O_4_) formula. However, it was shown that natural and synthetic moolooite crystals contain a significant portion of “structural” water, which cannot be ignored. Considering the substantially variable amount of water, which can be incorporated into the crystal structure, the formula Cu(C_2_O_4_)·*n*H_2_O for moolooite is justified.

## 1. Introduction

Oxalate minerals are quite a representative class of minerals, as more than two dozen of them are known to form when sources of oxalic acid interact with metal cations leached from primary minerals [1,2,3]. Acid-producing microorganisms (fungi, lichens and bacteria) as well as guano are common sources of oxalic acid [1,4,5,6,7]. Some of the oxalate minerals were discovered specifically as biominerals, such as lindbergite [8]. For most other natural salts of oxalic acid, the connection of their formation with the activity of biological organisms is very likely [9]. One of the best examples of oxalate biominerals is moolooite. Moolooite was discovered in 1986 as the result of the interaction of bird guano with chalcopyrite at Mooloo Downs Station in Western Australia [7]. In addition, findings of moolooite in lichens growing on copper-bearing minerals were described in the works by Purvis [10,11,12]. This is also confirmed by recent findings of moolooite in apothecia in saxicolous lichen *Lecidea inops* on weathered chalcopyrite ore at the Voronov Bor deposit (Central Karelia, Russia) in 2020 [2] and in *Lecanora polytropa* on volcanic rock samples from the Tolbachik volcano area (Kamchatka, Russia) in 2019 [13].

The study of the crystallization of copper oxalate is very interesting and promising, since copper is a toxic element and the formation of insoluble copper oxalate (moolooite) can be used in the bioremediation technologies for copper-contaminated environmental objects with the help of oxalate-producing microorganisms [4,14,15,16]. For example, Tsekova [17] described the recovery of copper and other metals from industrial wastewater using *Aspergillus niger*, which is one of the most commonly used models of oxalate-producing micromycetes [4,18,19]. Moreover, numerous publications on applications in industry due to the display of interesting physical and chemical properties, including antiferromagnetic ones [20,21], and due to it acting as a precursor to the formation of a number of widely used nanoparticles, such as Cu, CuO, Cu_2_O and Cu(OH)_2_ [4,22,23,24], can be found for copper oxalate.

The properties of copper oxalate have been studied for over five decades now, and it is not surprising that the amount of related research exceeds a couple dozens of papers. Approximately half of them discuss its possible crystal structure [25,26,27,28,29,30,31,32,33,34].

**Figure 1:**
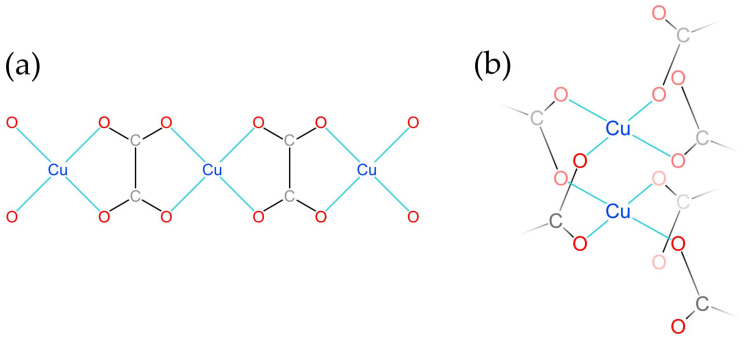
Fragments of two possible crystal structures of copper oxalate proposed by Dubicki et al. [24].

To the best of our knowledge, the first two possible structural models of copper oxalate were proposed by Dubicki et al. [25] after studying its magnetic properties. The first model consists of isolated chains of copper ions in a square-planar geometry connected successively via oxalate molecules (Figure 1a). The second model suggested the framework structure where each Cu^2+^ cation is connected to four oxalate molecules (Figure 1b).

Over the course of investigating the powder X-ray diffraction (PXRD) patterns of a set of synthetic Cu^2+^ oxalate samples, Schmittler [26] observed sample-to-sample differences in X-ray line shifts, the broadening of diffraction peaks and the presence of additional peaks in several samples. The study of PXRD patterns made it possible to propose a chain-based structural architecture (Figure 1a) as the most probable, and to calculate bond distances for the respective configurations. It is worth noting that the work by Schmittler was the first to propose the presence of structural disorder. Schmittler suggested that the structure of moolooite has the space group *Pmnn*, and that the disorder is probably caused by the alternation of two ordered phases. Schmittler also established a correlation between unit cell parameters and the H_2_O content, and related the presence of diffuse peaks on the PXRD patterns with the number of H_2_O molecules per formula unit. A decade later, data on different oxalates allowed Fichtner-Schmittler [27] to suggest the presence of the order–disorder (OD) character of copper oxalate. It was argued that the differences between the resulting PXRD patterns of CuC_2_O_4_∙*n*H_2_O can be attributed to the existence of two maximum-degree-of-order (MDO) polytype structures that share common family reflections.

Michalowicz et al. [28] investigated the X-ray absorption spectrum using the EXAFS technique and managed to calculate the Cu–O, Cu–C and Cu–Cu distances. The data from the EXAFS studies were evidence in favor of the chain-based structural architecture, and the results of both PXRD and EXAFS were discussed later by Fichtner-Schmittler [29]. Gleizes et al. [30] solved the structure of Na_2_Cu(C_2_O_4_)_2_∙2H_2_O, and based on its structure and the EXAFS data deduced that the structure of CuC_2_O_4_∙*n*H_2_O corresponds to the chain-based structural architecture (Figure 1a).

Kondrashev et al. [31] managed to synthesize both disordered and ordered phases of copper oxalate, and denoted the former and the latter as the α- and β-phase, respectively.

In 1977, a natural specimen of copper oxalate was discovered by Clarke and Williams [7]. The authors claimed that the specimen was formed by the interaction of solutions derived from bird guano and the weathering of copper sulfides. Soon after the first report, the same mineral was identified in lichen species growing in areas of copper mineralization [11]. PXRD patterns of both samples were indexed based on the orthorhombic unit cell described by Schmittler [26]. Both samples have similar unit cell parameters and calculated H_2_O contents, which are significantly lower than those in the synthetic material.

The influence of hydroxypropylmethylcellulose on the precipitation of copper oxalate was studied by Jongen et al. [32]. The resulting crystals were studied using PXRD, FT-IR spectroscopy and electron microscopy. It was noted that electron diffraction patterns demonstrate the presence of diffuse streaks at the *l* = 2*n* + 1 reciprocal planes, indicating disorder parallel to the [110] direction.

The first structure solution was provided by Christensen et al. [33] using data obtained via synchrotron PXRD and neutron powder diffraction. The structure was solved using ab initio methods in the *P*2_1_/*n* space group, and the model accounted for stacking faults and microstructural size and strain effects. The authors argued the H_2_O/D_2_O molecules are absent from their particular studied sample, according to the results of the in situ synchrotron PXRD thermal decomposition data, thermogravimetric analysis and neutron powder diffraction structure analysis.

The most recent data on the moolooite structure were published by O’Connor et al. [34]. The authors used synchrotron PXRD and refined the structure of natural and synthetic moolooite using the Rietveld method on the basis of the structural model proposed by Schmittler [26]. H_2_O content is a topic which deserves special attention. Using Schmittler’s linear correlation for the *a* and *b* parameters of the pseudo-orthorhombic unit cell, O’Connor et al. [34] calculated the H_2_O contents for all the published structural data and concluded that the amount of H_2_O in natural and synthetic samples may be attributed to the conditions under which moolooite crystals form.

Despite the results of the numerous studies discussed above, the problem with the moolooite crystal structure has not yet been resolved due to the absence of direct single-crystal studies.

Herein, we report on the preparation of a synthetic analog of moolooite (**SM**) and on the structural model of moolooite obtained for the first time from single-crystal X-ray diffraction data. In addition, the H_2_O content in the moolooite structure, which is also a matter of speculation nowadays, is discussed via the presentation of a conception of its impact on the size and stability of copper oxalate crystals.

## 2. Results

### 2.1. Infrared Spectroscopy

**Figure 2:**
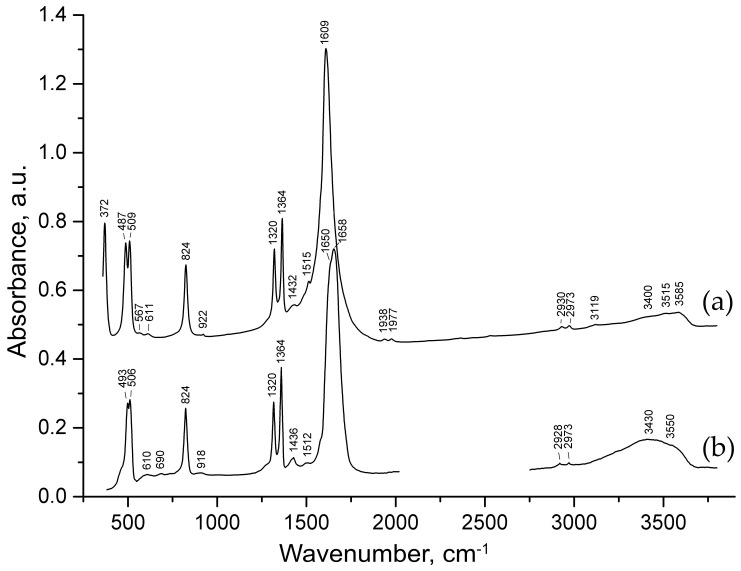
IR spectra of **SM** (**a**) and natural moolooite from Sarbay (**b**) [5].

The infrared (IR) spectra of the **SM** and natural moolooite [5] samples (Figure 2) are similar. The main distinctions are in the high-frequency range of 1600–3600 cm^−1^.

The band assignments are as follows: The range 3400–3600 cm^−1^ corresponds to O–H stretching vibrations of H_2_O molecules forming weak (>3500 cm^−1^) and relatively strong (<3500 cm^−1^) hydrogen bonds. Antisymmetric C–O stretching, symmetric C–O stretching, O–C–O bending and two kinds (in-plain and out-of-plane) of O–C–C bending vibrations correspond to the strong bands at 1609–1655, 1320–1364, 864, and 487–372 cm^−1^. The band at 690 cm^−1^ may be due to libration vibrations of H_2_O molecules. Weak bands of H–O–H bending vibrations (in the range of 1600–1700 cm^−1^) could not be observed because of the overlapping with a much stronger band of oxalate anions. Other very weak bands correspond to overtones and combination modes.

### 2.2. Single-Crystal X-ray Diffraction Analysis: Data Processing Details

Diffraction data obtained via the single-crystal X-ray diffraction experiment (see the Section 4 for details) were thoroughly analyzed using CrysAlisPro software [35]. The orthorhombic unit cell was the first and obvious choice for data processing: the automatic algorithm of CrysAlisPro proposed a unit cell with the following unit cell parameters: *a* = 5.601(2) Å, *b* = 5.415(3) Å, *c* = 2.5553(8) Å and *V* = 77.50(5) Å^3^. SCXRD data can be processed in an orthorhombic setting with good reliability values (*R*_int_ = 4.66% and *R*_σ_ = 3.30%). The structural model was solved and refined in the *Pnnm* space group up to *R*_1_ = 7.26%, *wR*_2_ = 20.27% and *S* = 1.135 for 129 unique reflections with |*F*_o_| ≥ 4*σF*. Despite relatively high convergence factors, the rest of the refinement parameters demonstrate a reliable structural model (Table 1, Supplementary Materials). Details on the crystal structure and the comparison with the available structural models are discussed below.

Although the structural model refined in the orthorhombic unit cell seems to be reasonable, twinning and diffuse scattering, which were observed within the experiment, were not taken into account.

**Figure 3:**
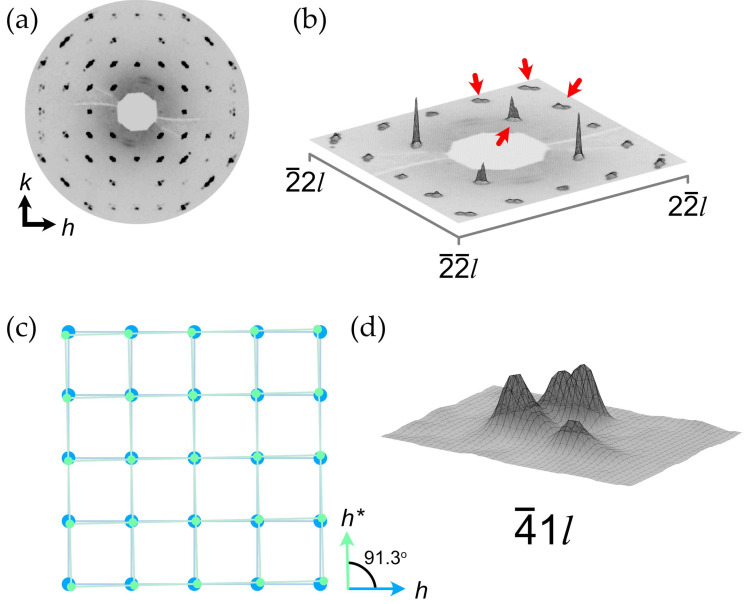
A precession image of the *hk*0 reciprocal lattice plane (**a**); 3D intensity vs. *hkl* plot of the *hk*0 lattice plane (**b**). Red arrows at (**b**) point out reflection twinning. There is an angular relationship between Bragg reflections of two twin domains (**c**); 3D intensity vs. *hkl* plot of the -41*l* reflection (**d**). Note: in order to improve the visibility of the diffraction pattern, an artificial scale factor has been applied during the reconstruction of precession images hereinafter.

Figure 3a provides a precession image of the *hk*0 lattice plane of diffraction data in the orthorhombic setting. The first important detail to notice is the presence of the splitting of reflections from the *hk*0 plane. There are two main twinning domains that are clearly seen at low 2*θ* angles (Figure 3b). Using a built-in algorithm in the CrysAlisPro software for twin component refinement, it was found that the second smaller domain is rotated at 91.3^o^ around in the [001] direction relative to the first domain (Figure 3c). The final structural model for both twin components (0.66(1):0.33(1) ratio) resulted in the worsening of the convergence factors (Table 1).

With the increase in the 2*θ* angle, additional satellite reflections start to appear more legibly (Figure 3d). The appearance of satellite reflections is probably due to the presence of additional domains that make a smaller contribution, and thus, their assignments were unsuccessful.

**Figure 4:**
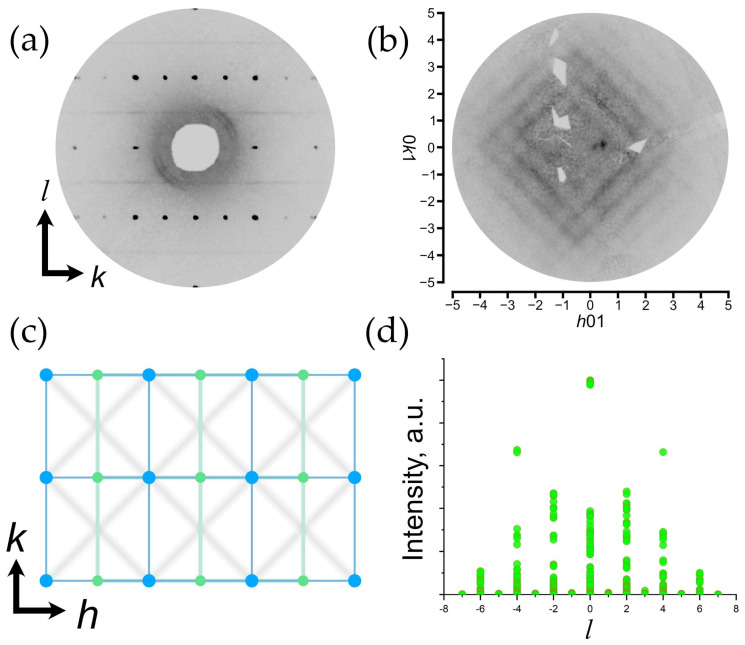
A precession image of the 0*kl* reciprocal lattice plane in orthorhombic setting (**a**); a precession image of the *hk*1 reciprocal lattice plane in monoclinic setting (**b**); a relationship between hypothetical Bragg reflections of orthorhombic (green) and monoclinic (blue) lattices overlapping diffuse scattering (gray) (**c**); a distribution of reflection intensity plotted vs. *l* values (**d**). Note: for (**b**), a background subtraction was applied with the minimum value set as background.

Another important moment is the presence of diffuse scattering at the *h*0*l* and 0*kl* lattice planes in the form of streaks located at the (00*l*) lattice planes where *l* = *n* + 0.5 (Figure 4a). As it was mentioned, the presence of diffuse lines and the associated doubling of the *c* parameter was observed in natural and synthetic samples earlier [7,26,31,32], which allowed for the proposal of the monoclinic setting of the unit cell accounting for the appearance of additional reflections. However, the automatic algorithm of the CrysAlisPro program failed to find the unit cell in the monoclinic setting due to the absence of sharp Bragg reflections. Thus, the unit cell parameters were calculated manually using the following trigonometric equations describing relationships between the unit cell parameters of orthorhombic and monoclinic settings [34]:

*a*_mon_ = *a*_orth_/cos(β − 90°), (1)

*b*_mon_ = *b*_orth_, (2)

*c*_mon_ = 2 × *c*_orth_, (3)

*β*_mon_ = 90° + tan^−1^(*c*_orth_/*a*_orth_). (4)

In a monoclinic setting, the condition of diffuse line manifestation is transformed to *l* = 2*n* + 1. The further description of diffraction pattern details will be given in terms of the monoclinic setting.

Figure 4b demonstrates the precession image of the *hk*1 lattice plane and reveals the fine structure of the observed diffuse scattering, which is manifested in the form of crossed streaks arranged according to the |*h*| + |*k*| = *n* + 0.5 (*n* = integer) rule in the orthorhombic setting and |*h*| + |*k*| = *n* (*n* = integer) in the monoclinic setting. The intensity maxima of crossed diffusion streaks are located at nodes and centers of unit cells with a monoclinic reciprocal lattice (Figure 4c). The lack of Bragg reflections with *l* = 2*n* + 1 hinders the processing of the diffraction data since the algorithm evaluates the peak findings during 3D profile analysis regardless of the user-defined peak table, and therefore, considers the slightest rise in intensity (diffuse scattering in our case) on the diffraction pattern as a possible reflection. Thus, the resulting *hkl* dataset contains a number of weak, meaningless reflections satisfying the *l* = 2*n* + 1 condition derived from the diffuse streaks, which is illustrated in Figure 4d with the intensity of reflections being plotted vs. the *l* value.

**Figure 5:**
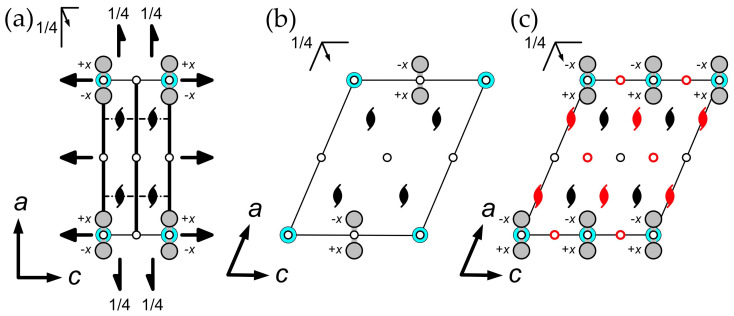
Projections of Cu and C atoms onto the (010) plane along the ±1/4*b* direction in (**a**) the **OP** model, (**b**) the perfectly ordered monoclinic model and (**c**) the disordered monoclinic model. For the sake of clarity, symmetry elements are superimposed over unit cell boundaries (thin black line) and atoms. Symmetry elements denoted in red in (**c**) are the result of the disorder occurrence (see text for details). Atoms in (**a**) and (**c**) are half-occupied. Legend: Cu = cyan; C = gray; O atoms are excluded for clarity.

The difference between the orthorhombic and monoclinic settings can be described by considering the crystal chemical nature in each case. As it was discussed above, the crystal structure of moolooite consists of infinite Cu–(C_2_O_4_)–Cu chains. In the hypothetical ordered structure, the length of the translation vector along the chain would be ~5 Å (as in the case of monoclinic setting), whereas the orthorhombic cell defines the translation vector along *c* to be equal to ~2.5 Å. Thus, the absence of Bragg scattering at |*h*| + |*k*| = *n* (*n* = integer) in the monoclinic setting indicates that there is clear evidence of the presence of a disorder along the direction of chain propagation. As it is shown in Figure 5a, the positions of Cu and C within the orthorhombic cell are disordered and half-occupied, and each Cu position is located at a C–C bond (this structural model will be referred to as **OP**).

Such a disorder affects the choice of origin in the monoclinic setting. In a perfect ordered structure, there are only two places for the inversion centers in the (0,0,0) and (0,0,1/2) positions to coincide with the Cu and the center of the C–C bond, respectively (Figure 5b). The disorder generates one more possible position at (0,0,1/4), which is located between the oxalate group and Cu atom in the ordered structure (Figure 5c). Thus, there are two possible options for the origin of the unit cell in the disordered monoclinic structure: the first origin is at (0,0,0), which is similar to the ordered structure with Cu atoms at the 2a and 2b Wycoff sites (**MC2**), whereas the second origin is at (0,0,1/4) with all the atoms at the 4e Wycoff sites (**MC4e**).

The final monoclinic structural model can be solved and refined using both origins of the unit cell. The **MC4e** model was refined in the *P*2_1_/*n* space group with the convergence factors of *R*_1_ = 9.58%, *wR*_2_ = 27.06% and *S* = 1.270 for 333 unique reflections with |*F*_o_| ≥ 4*σF* (Table 1). The **MC2** model was refined in the *P*2_1_/*n* space group with the convergence factors of = 13.24%, *wR*_2_ = 39.07% and *S* = 1.230 for 336 unique reflections with |*F*_o_| ≥ 4*σF* (Table 1). As it can be seen from the convergence factors, the **MC4e** model describes the experimental data much better. However, from the crystal chemical point of view, the **MC4e** is not correct due to the position of the inversion center as it cannot be placed between the oxalate group and Cu atom. Therefore, the **MC4e** model characterizes the statistically disordered structure, whereas the **MC2** model is more correct in a crystal chemical sense. It should be noted that the parameters influencing the choice of a particular origin of the unit cell have not been cleared up.

### 2.3. Crystal Structure Description

Despite the differences in the diffraction data, both orthorhombic and monoclinic models show the same structural architecture, which is in a good agreement with previously published studies. As we demonstrated above, the most prominent similarity between the models in both settings is the disorder of the Cu and C sites. Despite the presence of reflections with *l* = 2*n* + 1 in the diffraction dataset of the monoclinic model, the disorder is realized as it is in the conventional superposition orthorhombic model. However, the lowering of the symmetry leads to the doubling of the number of symmetrically nonequivalent positions of C and O.

**Figure 6:**
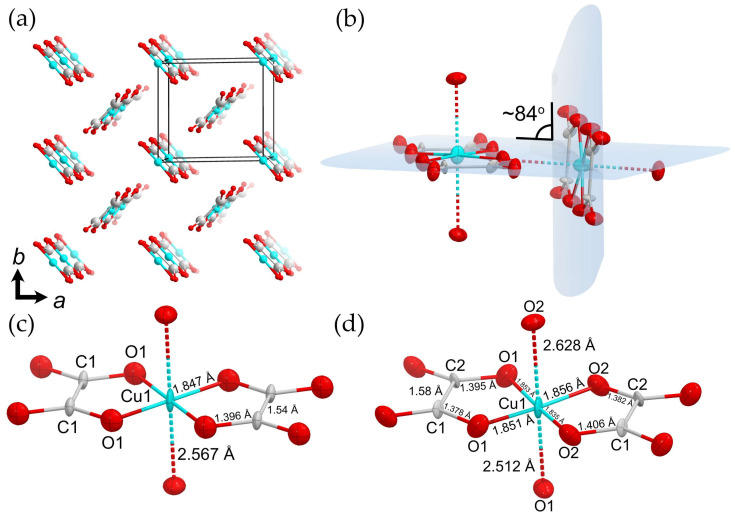
The crystal structure of copper oxalate (**a**); an illustration of chain inclination (**b**); a fragment of the copper oxalate chain in the orthorhombic setting (**c**); a fragment of the copper oxalate chain in the monoclinic setting (**d**). Thermal ellipsoids are drawn at 50% probability level. Legend: Cu = cyan; O = red; C = gray.

The fundamental building unit of the moolooite structure is the Cu–(C_2_O_4_)–Cu chain arranged along the [001] direction (Figure 6a). The chains are inclined at ~84^o^ to each other (calculated as the angle between mean planes drawn through O atoms of each chain) and form the infinite framework via elongated Cu–O bonds between adjacent chains (Figure 6b). On the other hand, Fichtner-Schmittler [27] described such stacking in terms of the OD theory, asserting that the chains connected via Cu–O form crossed layers that are coplanar with the (110) and ($1\overset{-}{1}0$) planes. Therefore, the overall structure can be described as a conditional “polytype”.

Each Cu atom is coordinated with four oxygen atoms belonging to the oxalate groups, where the Cu–O bond distances are 1.847 Å in the **OP** model (and <Cu–O> = 1.849 Å in the **MC4e** model), thus forming an equatorial plane. The Cu atoms form two additional long bonds with apical O atoms of neighboring chains with a Cu–O distance of 2.567 Å in the **OP** model (Figure 6c), while the bond lengths in the **MC4e** model are 2.512 and 2.628 Å (Figure 6d). Such strong anisotropy of the bond length distribution is caused by the Jahn–Teller effect, which is associated with the degenerate electronic ground state of a *d*^9^ metal in an octahedral ligand field [36,37,38]. As it was demonstrated by Burns and Hawthorne [39] and Kornyakov [40], the resulting [4+2]-elongated octahedron is the most common coordinate geometry for the crystal chemistry of copper oxysalts; however, the bond valence sum of copper of ~2.7 valence units (v.u.) shows its strong oversaturation. The possible cause of the Cu oversaturation is the libration effect expressed in the enlarged anisotropic displacement parameters of Cu and O atoms. This suggestion is supported by the C–O bond length of ~1.39 Å, which is ca. 0.15 Å longer than lengths of 1.24–1.25 Å observed in crystal structures of various oxalates [41,42,43]. The imaginary shifts of the O atoms towards the C atoms leads to the increase in the Cu–O bond lengths, resulting in the elongation of the Cu–O bond lengths. Another possible mechanism of the valence relaxation is a slight shift of the Cu atom out of the equatorial plane of the octahedron, leading to the elongation of Cu–O equatorial bonds, which is indirectly confirmed by the elongation of the thermal ellipsoid of Cu (Figure 6c,d). The C–C bond lengths are 1.54 and 1.58 Å in the orthorhombic and monoclinic models, respectively, which is consistent with the published crystal structures containing oxalate dianions [41,42,43].

In general, the resulting structural model agrees well with the previously published models. As shown in Table 2, the bond distance values are close to those published by Schmittler [26] and O’Connor et al. [34] on the basis of disordered structural models. The only exception is related to the C–C bonds, which are significantly longer compared to our data. On the other hand, our bond length values differ from those presented by Christensen et al. [33]. It can be suggested that such a discrepancy comes from the differences in the diffraction datasets—whole data from both superimposed “polytypes” in our case vs. split data from both crystallites found in the previous work [33].

### 2.4. Structural Disorder Modeling Based on the Diffuse Scattering Simulation

**Figure 7:**
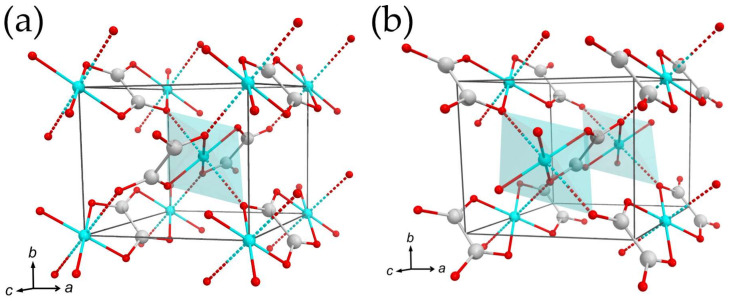
The crystal structures of the **MC2a** model (**a**) and **MC2b** model (**b**). Cu atoms at the center of the unit cells are showed within CuO_6_ octahedra. Legend: same as that in Figure 6.

As it was described above, the presence of diffuse scattering located at the *l* = 2*n* + 1 lattice planes (Figure 4) was detected in previous studies, and several structural models were proposed on the basis of powder diffraction data [26,27,31]. However, the data obtained via single-crystal X-ray diffraction make it possible to take a closer look at the nature of the observed disorder. In this section, we employ a computer simulation approach in order to reveal basic structural features causing the observed diffuse scattering. Prior to discussion, several assumptions should be defined:

- The crystal structure in the **OP** cell is not suitable for calculations due to its disordered nature. As it was mentioned above, the orthorhombic cell defines the translation vector along *c* to be equal to ~2.5 Å, which is only half of the shortest distance to maintain an order within the Cu–(C_2_O_4_)–Cu chain. Thus, calculations should be conducted on the basis of the monoclinic unit cell with full translation.
- The process of a diffuse scattering simulation demands the availability of a model demonstrating clear and visible disorder with split sites. The availability of the **MC4e** model satisfies these requirements with one important exception: the Wykoff site of Cu is 4e, which means that the structure contains a single symmetrically nonequivalent Cu site, and thus, the hypothetical ordered model cannot be obtained. On the other hand, the **MC2** model shows a clear splitting of the Cu and C positions into two sets, which makes it possible to conduct calculations based on two hypothetically ordered models: the **MC2a** contains Cu atoms at the vertices and the center of the unit cell (the 2a Wyckoff sites; see Figure 7a), whereas the Cu atoms in the **MC2b** are arranged at the center of the *c* edge (0,0,1/2) and in the center of the *ab* face (1/2,1/2,0) of the unit cell (the 2b Wyckoff sites; see Figure 7b).
- Several features complicating calculations should be taken into account. First of all, both **MC2a** and **MC2b** are still based upon diffraction data obtained from the crystal formed by two superimposed “polytypes”, which is expressed in nonconventional bond lengths (see Section 2.3). Secondly, the diffraction intensity of diffuse scattering is too low to establish reasonable intensity vs. *hkl* correlations (the period of intensity variation). Additionally, the presence of twin domains, as described above, can possibly affect the observed diffuse scattering. Thus, the goal of the calculations is to confirm the origin of the disorder and to simulate the general diffraction pattern.

Calculations were conducted using DISCUS software implemented via the DISCUS Suite program package [44,45,46]. The simulating mechanism was based upon direct Monte Carlo calculations. Calculations were conducted using the energy coefficient *J_n_*, which stands for interaction energies, where the value of 1.0 means that the desired Monte Carlo move is energetically unfavorable and −1.0 means that a move is energetically favorable. Although we use energy as the definition of the desired Monte Carlo moves, the final result is given in correlation coefficients for the sake of clarity. Detailed descriptions of the method can be found in Refs. [44,47,48]. In order to simplify the simulation process, the origins of unit cells of the **MC2** models were shifted using the 0.25*a* + 0.25*b* + *c* vector. A final simulated precession image of the *hk*1 lattice plane was drawn on the basis of a constructed fragment of a 2D crystal of 500 × 500 × 1 unit cells. In order to obtain a smooth noiseless image, Fourier calculations were conducted using 100 lots (randomly chosen subregions of 50 × 50 × 1 unit cells) assuming the periodic boundary condition. The *h*0*l* image was obtained on the basis of 200 × 40 × 200 unit cells and 100 lots of 20 × 20 × 40 unit cells. Bragg scattering was determined as 1% of the crystal subtracted from the resulting images in both cases. The resulting precession images, as well as the schematic illustrations of the crystal representing the used correlation vectors, were drawn using KUPLOT software [46].

As it can be seen from Figure 4b, the observed diffuse scattering at the *hk*1 lattice planes consists of two crossed sets of diffuse streaks: **H** ± $\epsilon \langle 1\overset{-}{1l}\rangle$* and **H** ± $\epsilon \langle 11l\rangle$* with *l* = odd (where **H** = (*h*, *k*, *l*) is a parent Bragg reflection and ϵ represents a continuous variable). The origin of the crossing of streaks is not clear and can be attributed to the intrinsic consequence of the particular disorder correlations within the crystal structure or to the twinning manifestation. However, the second twin domain is much smaller than the dominant one (0.66(1):0.33(1)), which is expressed in the BASF parameter (Table 1) and the reflection intensity distribution (Figure 3b). Considering the overall low intensity of the diffuse scattering, we will further assume that crossed diffuse streaks are the consequence of the disordered nature of the studied crystal. However, the influence of the second twin domain cannot be excluded, and due to this fact, the overall intensity of the observed diffuse scattering cannot be taken into account.

**Figure 8:**
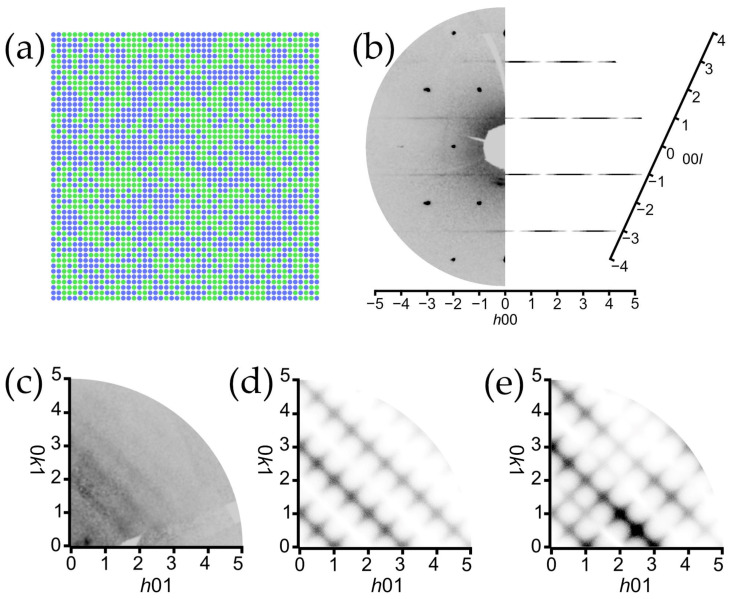
Simulated array of unit cells (50 × 50) demonstrating the result after correlation vectors applied (see text for details) (**a**); comparison between the reconstructed observed (left) and simulated (right) *h*01 reciprocal lattice planes (**b**); the first quadrant of the reconstructed precession image of the *hk*1 lattice plane (**c**); the first quadrant of a simulated *hk*1 reciprocal lattice plane according to the structural model proposed in the present study (**d**); the first quadrant of a simulated *hk*1 reciprocal lattice plane calculated using structural models proposed by Christensen et al. [33] (**e**). Circles in (**a**) are unit cells of **MC2a** (denoted blue) and **MC2b** (denoted green) hypothetical structural models.

Since the Cu and C sites in the refined **MC2** structural model are each 50% occupied, the starting point of the simulating process is a 50% random distribution of the **MC2a** and **MC2b** unit cells. The simulation of the *hk*1 diffraction pattern using a random unit cell distribution gives smooth, continuous diffuse streaks running along the **H** ± $\epsilon \langle 11l\rangle$* with *h* + *k* = odd, which is already quite close to the observed patterns. It is obvious that the correlation with **H** ± $\epsilon \langle 1\overset{-}{1l}\rangle$* is required to obtain the second set of streaks. After several attempts to model a disordered crystal structure, the following correlation vectors were chosen (where each digit addresses the desired unit cell relative to the origin cell): (i) vectors $\langle \left|1\right|0\rangle$ and $\langle 0|1|\rangle$; (ii) vectors $\langle \left|1\right||1|\rangle$; and (iii) vectors $\langle \left|2\right|0\rangle$ and $\langle 0|2|\rangle$. Only the second vector represents the desired correlation within the crystal, whereas the first and the third sets serve as limits to the second one. The resulting correlation values are 0.0 (the correlation is absent), 0.3 (moderate correlation) and 0.0 for each set of vectors. Figure 8a shows the crystal modeled using the correlation vectors given above and their respective values, where circles represent unit cells and each ordered unit cell is marked by its own color (blue = **MC2a** and green = **MC2b**). As it can be seen, the correlation of the disorder is not limited to diagonal directions only, and provides a significant degree of freedom for possible stacking faults: the lengths of faults vary from several unit cells to dozens of them, which allows characterizing it as nanoscale structural disorder. Simulated diffraction images are shown in Figure 8b,d. An observed diffuse scattering is provided in Figure 8c. It should be noted that both sets of diffuse streaks are present (Figure 8d). However, the **H** ± $\epsilon \langle 11l\rangle$* set of streaks is still manifested at *h + k* = 2*n* + 1 only, whereas the **H** ± $\epsilon \langle 1\overset{-}{1l}\rangle$* set does not show the same distribution order. In other words, the extinction condition *h* + *k* + *l* = 2*n* (along with the *h* + *k* = 2*n* + 1 and *l* = 2*n* + 1) characterizes the **H** ± ϵ〈11l〉* set of diffuse streaks, whereas the **H** ± $\epsilon \langle 1\overset{-}{1l}\rangle$* set has only the *l* = 2*n* + 1 condition. We assume that such a diffuse intensity distribution is mainly dependent on the particular extinctions associated with reflection from the corresponding sublattices within the resulting crystal model. As it was mentioned, the incorporation of a Cu–C disorder results in the appearance of **H** ± $\epsilon \langle 11l\rangle$* of diffuse streaks. Thus, the set of diffuse streaks can be described by the combination of reflection conditions for sublattices formed by particular atom sites. Let us consider these reflection conditions (note that only conditions relevant for the *hk*1 plane are taken into account) [49]:

- The Cu atoms of both models are located at the 2a and 2b Wyckoff sites with sublattices, satisfying the following reflections conditions: *h* + *k* + *l* = 2*n* and *h* + *l* = 2*n* (on the *h*0*l* plane);
- The C atoms are located at 4e Wyckoff sites and have only one meaningful condition: *h* + *l* = 2*n* (on the *h*0*l* plane);
- The O atoms are also located at 4e Wyckoff sites with the same condition: *h* + *l* = 2*n* (on the *h*0*l* plane).

**Figure 9:**
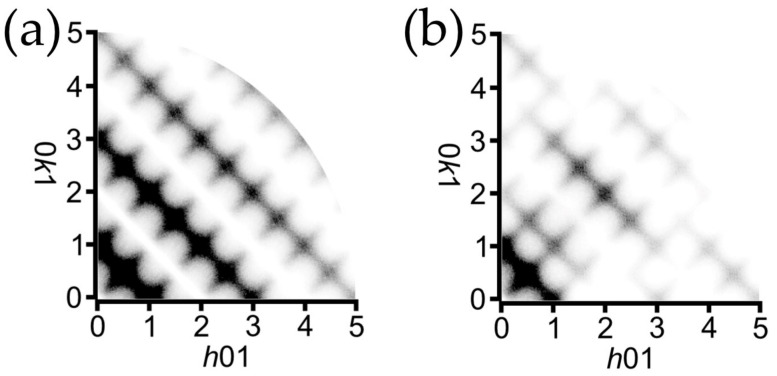
*hk*1 plane calculated using structures obtained via Monte Carlo simulations: calculated including Cu atoms only (**a**); calculated including C atoms only (**b**). Intensities for C atoms enhanced 10 times with respect to the intensities for Cu atoms.

Thus, sublattices formed by C and O atoms should contribute to the *h* + *k* = 2*n* + 1 scattering, which is absent in Figure 8d. It is of interest that the *h* + *k* = 2*n* streaks related to the scattering caused by the sublattice formed by C atoms are actually present, but the resulting intensity is too low compared to that of the scattering from Cu atoms (see Figure 9). However, the intensity of the *h* + *k* = 2*n* streaks should be amplified by the scattering from the sublattice formed by O atoms, which occupy general sites as well, and which is not observed in the simulated pattern. The most likely explanation is that O atoms form an ordered sublattice, which contributes to the Bragg scattering only. This assumption is confirmed by the fact that O atom sites in both the **MC2a** and **MC2b** models are the same, and thus, O atoms are ordered throughout the whole structure. If the same simulation is made using the models suggested by Christensen et al. [33], in which O atoms occupy different sites in both models, weak *h* + *k* = 2*n* streaks are clearly evident at *h* + *k* = 2 and *h* + *k* = 4, but the crossing of both sets of diffuse streaks is observed at (0.5, 1.5, *l*), (1, 1, *l*) and (1.5, 2.5, *l*) (*l* = 1) (Figure 8e). Thus, we can conclude that the presence of *h* + *k* = 2*n* streaks at the observed diffraction patterns (Figure 4b and Figure 8c) depends on the combined disorder of sublattices formed by C and O atoms. At the same time, the whole set of **H** ± $\epsilon \langle 1\overset{-}{1l}\rangle$* streaks reflects the presence of disorder, which is caused by the disorder of sublattices in combination with the correlated disorder along the $\langle 1\overset{-}{1l}\rangle$* vector.

Thus, the analysis of diffuse scattering allows one to figure out the fine features of the disorder within the moolooite crystal structure. The principal cause of the disorder is the shift of Cu oxalate chains relative to each other and as they lengthen, which leads to the appearance of diffuse scattering and the **H** ± $\epsilon \langle 11l\rangle$* set of diffuse streaks in particular. The disorder is moderately correlated at a nanoscale structural level, which results in the **H** ± $\epsilon \langle 1\overset{-}{1l}\rangle$* set of diffuse streaks. However, the simulated diffraction pattern does not completely replicate the observed one: the *h* + *k* = 2*n* diffuse streaks of the **H** ± $\epsilon \langle 11l\rangle$* set are still too weak (Figure 8c–e). It should be noted that diffuse scattering is absent in the *h*01 and 0*k*1 rows with *h* and *k* = 2*n* within the simulation experiment, which is most likely caused by the breaking of the local symmetry.

## 3. Discussion

### 3.1. Thermal Behavior

**Figure 10:**
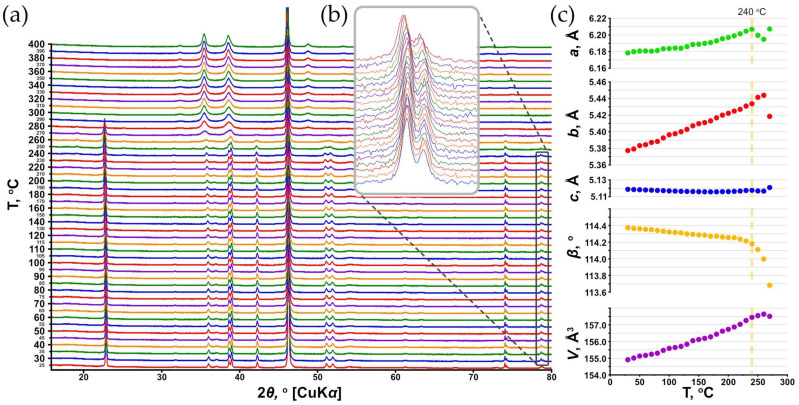
Powder X-ray diffraction patterns (**a**) and the unit cell parameters of **SM** as a function of temperature (30–400 °C) under heating in air (**b**). The 11-4 reflection displacement in a temperature range of 30–230 °C with a step of 10 °C (**c**). ESDs of the unit cell parameters are within the limits of the symbols.

The high-temperature PXRD experiment demonstrated the presence of the only **SM** phase (PDF 00-089-2531; ICDD PDF-2 Database, release 2019 [50]), which remains stable under continuous heating up to 250 °C. Later, starting at ca. 260 °C, the crystallization of the CuO phase, which is an analog of tenorite (PDF 00-021-0297 [51]), begins. The PXRD pattern obtained at 290 °C contains only peaks of CuO, while peaks related to **SM** disappeared (Figure 10a). However, it should be noted that unit cell parameters at 250 °C significantly stopped following the trend; thus, the calculations of the main coefficients of the thermal expansion tensor were carried out up to 240 °C.

Figure 10a illustrates the behavior of the unit cell parameters of **SM** as a function of temperature. Equations describing the temperature dependence of the unit cell parameters of **SM** within the range of 30 to 240 °C (Figure 10b) are: *a* = 5.62 × 10^−7^ × *T*^2^ − 2.51 × 10^−5^ × *T* + 6.180; *b* = 6.32 × 10^−8^ × *T*^2^ + 2.51 × 10^−4^ × *T* + 5.369; *c* = 1.91 × 10^−7^ × *T*^2^ + 6.08 × 10^−5^ × *T* + 5.121; *β* = −1.32 × 10^−6^ × *T*^2^ − 4.25 × 10^−4^ × *T* + 114.38; and *V* = 2.38 × 10^−5^ × *T*^2^ + 5.19 × 10^−3^ × *T* + 154.77.

**Figure 11:**
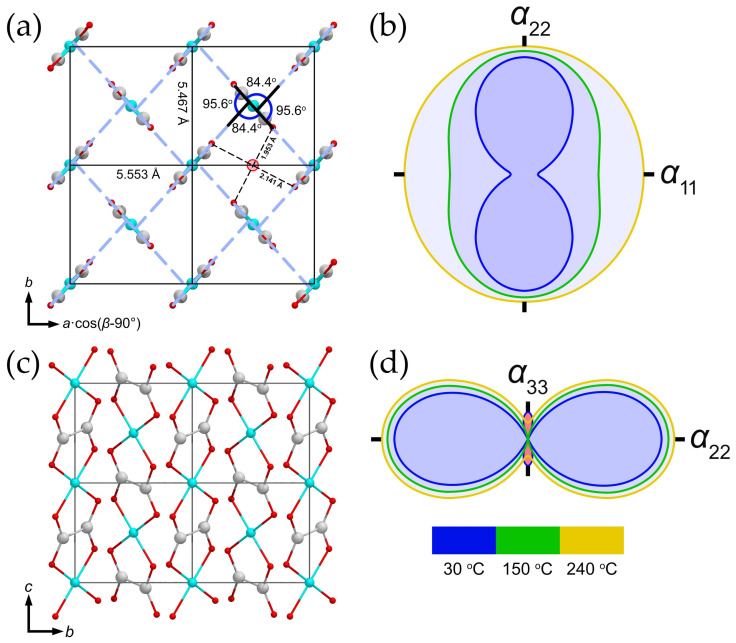
Crystal structure of **SM**, projections along [001] (**a**) and [100] (**c**); the respective arrangement of the figures of thermal expansion/contraction coefficients (TECs) in the structure of **SM** (**b**,**d**). Large red sphere at the center of the unit cell’s edge corresponds to the hypothetical site of H_2_O molecule, arranged in the center of the channel (see Section 3.2 for details). Legend: same as that in Figure 6; figures of TEC: expansion = blue and grey fill, contraction = red fill.

The thermal behavior of **SM** is essentially anisotropic (Figure 11, Table 3). The maximal thermal expansion of the crystal structure of **SM** is observed along [010] (Figure 11a,b). It is of interest that the section of the unit cell that is perpendicular to the [001] direction in the structure of **SM** is quite symmetrical and close to a square (Figure 11a). The possible higher symmetry reduction is expressed in various inclination angles between equatorial planes of Cu oxalate chains. Thus, the maximal thermal expansion of **SM** coincides with a larger (obtuse) angle between equatorial planes of chains, and with the smallest edge of the section. The thermal expansion coefficient (TEC) along [010] uniformly and rather weakly increases within the whole temperature range, while ***α*_11_** TEC, which is conditionally co-directional with [100], has low values at low temperatures and significantly increases with heating, and thus, the thermal expansion at temperatures closer to the **SM** stability limit within the (001) plane is nearly isotropic (Figure 11b). This can be explained by the tendency of the crystal structure to realize its higher possible symmetry, thus making the section perpendicular to [001] closer to a square (increase the *b* parameter to the value of *a*·cos(*β*-90°), and make the angles between the equatorial planes of chains closer to the orthogonal values). Further, another interesting feature of the **SM** crystal structure’s thermal behavior is the negative thermal expansion, which occurs approximately along [001] at the start of heating until approximately up to 160 °C (Figure 11c,d); afterwards, weak positive expansion is observed to be co-directional with Cu oxalate chain elongation.

The thermal decomposition of salts of oxalic acid could be dependent on the atmosphere in the furnace. For example, carbon monoxide oxidation during the first decomposition step of the calcium oxalate shifts to lower temperatures and allows us to determine the chemical composition accurately [52,53]. However, according to the data obtained for **SM** samples, there is no significant change in the decomposition steps’ resolution in measurements in different atmospheres (see Section 4.1 and Section 4.5).

The thermal decomposition of salts of oxalic acid could be dependent on the atmosphere in the furnace. For example, carbon monoxide oxidation during the first decomposition step of the calcium oxalate shifts to lower temperatures and allows us to determine the chemical composition accurately [52,53]. However, according to the data obtained for **SM** samples, there is no significant change in the decomposition steps’ resolution in measurements in different atmospheres (see Section 4.1 and Section 4.5).

**Figure 12:**
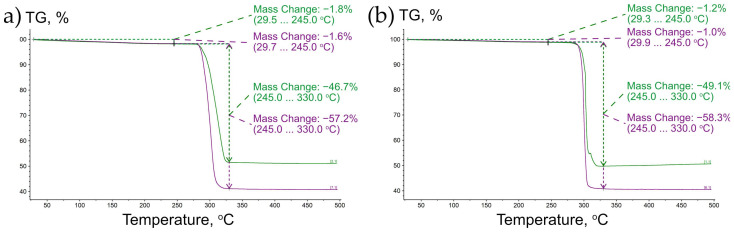
Thermal decomposition of **SM** powder samples that were synthesized at room temperature (**a**) and at 60 °C (**b**); see Section 4.1 for details. Legend: heating in inert (violet) and oxidizing (green) atmosphere.

The thermal decomposition of both measured samples occurs in two main steps (Figure 12) and fully corresponds to the results of the high-temperature PXRD experiment. The first step occurring in the temperature range of 30–245 °C is attributed to water elimination. There is no difference in sample behavior in different atmospheres at this step, as was expected. The second step occurring from 280 °C to 330 °C is related to (C_2_O_4_)^2−^ decomposition. Similar to other oxalates, under the oxidizing conditions the process finishes at lower temperatures than when carried out in the inert atmosphere [1,54,55,56,57], but there is no opportunity to split this step into smaller ones accurately. It should be noted that under inert conditions, the residual composition could include: carbon and Cu(I) and Cu(II) oxides. The final product for both samples obtained in a synthetic air atmosphere was identified as CuO.

### 3.2. H_2_O Content in the Structure of Moolooite

One of the most important issues regarding moolooite, along with defining its structural architecture, is the water content in its crystal structure.

The IR spectrum of natural moolooite (Figure 2b) differs from that of its synthetic counterpart due to a larger amount of H_2_O molecules forming strong hydrogen bonds, a significant shift in the band of antisymmetric C–O stretching vibrations and the presence of a band of H_2_O libration vibrations. A significant shift in the band of antisymmetric C–O stretching vibrations (of 49 cm^−1^) observed in the IR spectrum of high-hydrous natural moolooite relative to the spectrum of the synthetic sample cannot be explained by a simple overlapping with the band of H–O–H bending vibrations because the intensity of the latter is low. Such a strong band shift may be a result of the resonance of antisymmetric C–O stretching and H–O–H bending vibrations, which would be possible only if H_2_O molecules that form relatively strong hydrogen bonds are a part of the crystal structure of moolooite. The shoulder at 1650 cm^−1^ in the IR spectrum of natural moolooite may be due to resonance splitting. Bands with wavenumbers > 3500 cm^−1^ may correspond to adsorbed water. The absence of additional bands (except for those attributed to H_2_O) indicates the purity of natural moolooite. The natural doublet at 493 and 506 cm^−1^ is less resolved. This is apparently due to the correspondence of these bands to vibrations with larger amplitudes, which are more sensible to the adsorbed water.

Considering the mass losses during thermal decomposition, the chemical formulae of the initial **SM** samples studied using the TG technique could be considered as follows: Cu(C_2_O_4_)_1.02_·0.15H_2_O for Sample 1 and Cu(C_2_O_4_)_1.05_·0.10H_2_O for Sample 2 (see Section 4.1 for details). It should be noted that during the thermogravimetry studies, no substantial mass loss (0.25–0.6%) was observed in the range of 30–100 °C (Figure 12), which indicates that almost all H_2_O should be regarded as “structural”, but not as “sorption” water.

The structural model, in which all oxygen atoms are localized as components of oxalate groups, demonstrates the absence of positions for possible H_2_O molecule assignment. The crystal structure of **SM** contains channels arranged along [001] between the Cu oxalate chains with the centers at 0.5*a* and 0.5*b* (Figure 11a). However, these channels are too narrow and do not contain any significant portion of residual electron density. In addition, distances between the hypothetical O atom arranged within the channel to O atoms of the oxalate groups from the neighboring Cu oxalate chains, with which supposed H-bonds should be formed, are too short and do not exceed 2.2 Å (Figure 11a). These observations are confirmed by the calculation of the void volume in the described structural model. Thus, the calculated void volume of a single channel in the unit cell (Figure 11a) is equal to ca. 1.3 Å^3^ (which corresponds to ca. 2.6 Å^3^ per unit cell), whereas a mean effective H_2_O molecular volume is 24(2) Å^3^ [58]. On the other hand, thermal and spectroscopic studies unambiguously indicate that there is rather significant H_2_O content in the moolooite structure.

Thus, the most reasonable explanation regarding an arrangement of H_2_O molecules in the structure of **SM** can be due to the structural disorder. As modeling experiments to account for the diffuse scattering have shown, there is no substantial contribution of the disorder along the [100] and [010] directions, whereas the most significant contribution comes from the [001] direction. Since the hypothetical incorporation of H_2_O molecules in the aforementioned narrow channels should result in significant stretching within the (001) plane, such a mechanism cannot be considered to be the main mechanism. However, there are significant stacking faults of Cu oxalate chains observed as they lengthen. Together with the fact that the equatorial plane of the Cu-centered octahedron has very similar dimensions to those of the oxalate group, which makes such a displacement of chains in terms of the symmetry possible, one can expect an additional type of disorder. This disorder is realized when chain breaks occur, followed by the appearance of vacancies instead of Cu atoms and/or oxalate groups, but with the preservation of periodicity along the *c* axis. Depending on the necessity to maintain both local and general charge balances, such gaps can contain either OH^−^ groups or H_2_O molecules. In both cases, H-bonds can be formed with O atoms of adjacent chains and with O atoms located on the other side of the break within the same chain. This assumption corresponds very well with the results of the HTXRD experiment, which demonstrates the negative thermal expansion of the structure of **SM** along the [001] direction within the temperature range of 30–160 °C (Figure 11, Table 3). The dynamics of the thermal behavior of **SM** are well illustrated by the change in the position of the reflections that are sensitive to the variation in the *c* unit cell parameter, for example, the 11-4 reflection (Figure 10c). Up to 160 °C, the 11-4 reflection shifts to a high angular region, which corresponds to a decrease in the *c* parameter; afterwards, the reflection begins to shift in the opposite direction, i.e., towards low angles. Since there are no geometric or other obvious reasons for the contraction of the structure along this direction, which corresponds to the strongest bonding within the structural model, it can be attributed to the release of H_2_O molecules arranged in the Cu oxalate chain breaks. After the release of H_2_O molecules from these gaps (after 160 °C), the crystal structure of **SM** undergoes classical thermal expansion: the lowest expansion is observed along the strongest bonding (i.e., along the Cu oxalate chains).

## 4. Materials and Methods

### 4.1. Synthesis

To obtain a synthetic analogue of moolooite, experiments were carried out at various temperatures and with a variable ratio of initial components. The crystalline phase of **SM** was obtained by precipitation from a 500 mL aqueous solution of copper chloride (CuCl_2_·2H_2_O, 98% Vekton, sample weight ranges between 0.17–1.37 g) and sodium oxalate (Na_2_C_2_O_4_, 99% Vekton, ranges between 0.13–1.0 g). Synthetic experiments were conducted at room temperature (23–25 °C) and in a drying cabinet at 60 °C. The pH of the initial solution was adjusted by the addition of small amounts of sodium hydroxide or hydrochloric acid solutions. The ratio of the initial concentrations of copper cations and oxalate ions was 1:1, 4:1 or 1:4, and the pH value of the solution varied from 2.0 to 10.0 (the exposure time of the solutions ranged from 7 to 45 days). After precipitation of a crystalline powder, it was filtered, washed with distilled water, and dried at room temperature. The yield of **SM** varied, and in several experiments reached 50%. Phase analysis was controlled via the PXRD method. Single crystals of rather good quality and size suitable for single-crystal XRD studies were obtained in two experiments: Sample 1—at room temperature with a concentration ratio of copper cations and oxalate ions equal to 4:1, a solution pH of 4.7–5.0 and an exposure time of 36 days; Sample 2—at a temperature of 60 °C, with a concentration ratio of copper cations and oxalate ions of 1:1, a pH = 2.0 and an exposure time of 35 days. Single crystals from both experiments were checked on the diffractometer and a crystal of better quality was selected from the 1^st^ experiment for further processing.

Both synthesized samples were pelletized and examined using a Hitachi S-3400N scanning electron microscope equipped with an AzTec Energy X-Max 20 spectrometer, with an acquisition time of 60 s per point (acceleration voltage 20 kV, beam current 2 nA) and processed via the Oxford AzTec software with the TrueQ technique. The chemical analyses showed that both samples did not contain any elements above the detection limit except for Cu, O and C.

### 4.2. Single-Crystal X-ray Diffraction

**Figure 13:**
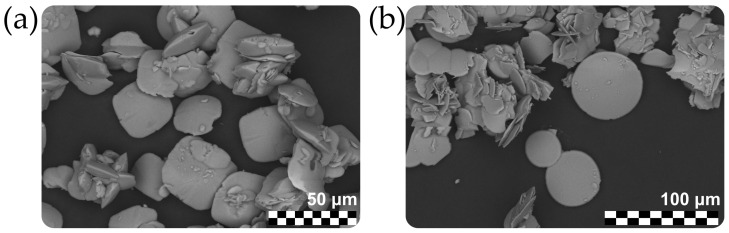
SEM images of **SM** crystals.

Crystals of **SM** have a lenticular shape and significantly vary in size, ranging from a few μm up to 0.1 mm in diameter (Figure 13). However, further single-crystal X-ray diffraction analyses showed that most crystals are not single. Some of them are multicomponent twins with a substantial contribution of a disordered structure, which results in intense diffuse scattering, and for others no Bragg reflections were observed.

A crystal of **SM** that was visually identified under an optical microscope in polarized light as being single was selected, coated in oil-based cryoprotectant and mounted on a cryoloop. The diffraction data were collected using a Rigaku XtaLAB Synergy S X-ray diffractometer operated with a monochromated microfocus MoKα tube PhotonJet-S (λ = 0.71073 Å) at 50 kV and 1.0 mA and equipped with a CCD HyPix 6000HE hybrid photon-counting detector [59]. The frame width was 1.0^o^ in ω, and there was a 180 s count time for each frame. CrysAlisPro software [35] was used for the integration and correction of diffraction data for polarization, for background and Lorentz effects, and for an empirical absorption correction based on spherical harmonics implemented in the SCALE3 ABSPACK algorithm. The unit cell parameters were refined using a least-squares technique. The structure was solved by a dual-space algorithm and refined using SHELX programs [60,61], which were incorporated in the OLEX2 program package [62].

In order to assess a possible disposition of H_2_O molecules, void spaces were calculated using the procrystal electron density method implemented in the *CrystalExplorer* software (version 21.5; the isovalue of 0.002 au was used) [63,64].

### 4.3. High-Temperature Powder X-ray Diffraction Studies

A crystalline powder sample of the synthetic Cu oxalate, which is an analog of moolooite (**SM**), was ground with an agate mortar for in situ examination using a Rigaku Ultima IV powder X-ray diffractometer (PXRD, CuKα radiation; 40 kV/30 mA; Bragg-Brentano geometry; PSD D-Tex Ultra detector; Tokyo, Japan). A Rigaku SHT-1500 chamber was used for data collection in air within a range of 25–400 °C with the *T* steps of 5 (25–120) and 10 °C (120–400). The heating rate was 2 °C/min. The collection time at each temperature step was about 30 min. A powder sample acting as a heptane suspension was placed on a Pt strip (20 × 12 × 1.5 mm^3^) that was used as the heating element and sample holder. The zero-shift parameter was refined at every step, and it was usually increased by 0.01–0.02° 2θ because of the sample holder expansion upon heating. The irreversibility of the observed phase transformations was verified by collecting PXRD data upon cooling. Phases were identified using the ICDD PDF-2 Database (release 2019; Newtown Square, USA). The unit cell parameters were calculated at every temperature step with the Rietveld method using the Topas 5.0 program package [65].

The main coefficients of the thermal expansion tensor were determined using a second-order approximation of temperature dependencies for the unit cell parameters by means of the TEV program [66]. The TEV package was also used to determine the orientation of the principal axes of the thermal expansion tensor and for visualization purposes.

### 4.4. Infrared Spectroscopy

In order to obtain the IR absorption spectrum of **SM**, the powdered sample was mixed with KBr that was predried at 120 °C, formed into a transparent pellet by pressurizing the mix at 10 tons in a hydraulic jack for 3 min and analyzed using an ALPHA FTIR spectrometer (Bruker Optics) with a resolution of 4 cm^−1^. A total of sixteen scans were obtained for each spectrum. The IR spectrum of a pellet of pure KBr was used as a reference blank.

The IR spectrum of natural moolooite from the Sarbay Fe deposit, Kostanay region, Kazakhstan, was obtained using a Specord 75 IR two-beam spectrophotometer (Carl Zeiss, Jena, Germany) with Slit Program 2. The procedure of sample preparation was the same as for **SM**. During the spectrum registration, a pure KBr pellet was placed in the reference beam.

### 4.5. Thermogravimetry Studies

Thermogravimetry studies on products of both successful experiments (Samples 1 and 2, see Section 4.1), in which single crystals of **SM** were obtained, were carried out in alumina crucibles in an inert (Ar) and oxidizing (80 N_2_/20 O_2_ synthetic air) atmosphere using the Netzsch TG 209 F1 Libra instrument. The samples’ masses were between 5 and 7.5 mg, and the temperature program steps included a 10 K/min heating step up to 500 °C, an isothermal hold for 10 min and further controlled cooling to reach room temperature.

## 5. Conclusions

In this paper, we reported on the crystal structure of the synthetic analog of the copper oxalate biomineral moolooite, which we refined from direct single-crystal X-ray diffraction studies for the first time. As it was shown, the refinement of the structure in the monoclinic setting allowed us to distinguish several fine details unavailable in the orthorhombic setting due to the averaging of atomic sites. The SCXRD experiment showed the substantial contribution of diffuse scattering to the overall diffraction data, which was caused by the nanostructural disorder along the strongest set of covalent bonds within the Cu oxalate chains. The description of the crystal structure indicates that the ideal structure of the Cu oxalate biomineral moolooite should not contain H_2_O molecules and should be described by the Cu(C_2_O_4_) formula. However, it was shown that natural and synthetic moolooite crystals contain a significant portion of “structural” water, which cannot be ignored. The combination of various experimental techniques allowed us to suggest the mechanism of H_2_O molecule incorporation in breaks of Cu oxalate chains. Moreover, this incorporation of H_2_O molecules apparently plays a significant role in the structural disorder, which can be also attributed to the absence of good-quality crystals in nature and in synthetic experiments: the more H_2_O content in the structure, the stronger the disorder will be, which significantly reduces the quality of single crystals. Considering the substantially variable amount of water, which can be incorporated into the crystal structure, the formula Cu(C_2_O_4_)·*n*H_2_O for moolooite is justified.

## Figures and Tables

**Table 1:** Crystallographic data, data collection and refinement parameters of **SM**.

Description	Orthorhombic Setting	Orthorhombic Setting with Twin Domain Accounted for	Monoclinic Setting (MC4e)	Monoclinic Setting (MC2)
*Crystallographic Data*				
Space Group	*Pnnm*	*Pnnm*	*P*2_1_/*n*	*P*2_1_/*n*
*a* (Å)	5.601(2)	5.607(2)	6.147(3)	6.111(4)
*b* (Å)	5.415(3)	5.428(2)	5.433(2)	5.467(3)
*c* (Å)	2.5553(8)	2.5579(7)	5.1173(18)	5.114(2)
*β* (^o^)	90	90	114.51(5)	114.68(7)
V (Å^3^)	77.50(5)	77.85(5)	155.51(12)	155.21(18)
Z	1	1	2	2
*Data Collection Parameters*				
Angle range 2*θ* (^o^)	10.476–65.388	10.456–65.894	8.764–66.906	8.778–66.536
Index range	−8 ≤ *h* ≤ 6, −8 ≤ *k* ≤ 6, −3 ≤ *l* ≤ 3	−8 ≤ *h* ≤ 8, −8 ≤ *k* ≤ 6, −3 ≤ *l* ≤ 3	−7 ≤ *h* ≤ 9, −8 ≤ *k* ≤ 7, −7 ≤ *l* ≤ 7	−9 ≤ *h* ≤ 7, −8 ≤ *k* ≤ 7, −7 ≤ *l* ≤ 7
Total reflections	753	382	1583	1538
Unique reflections	169	382	515	512
Reflections with *F*^2^ > 2σ(*F*^2^)	129	316	333	336
*R*_int_, *R*_σ_ (%)	4.66, 3.30	*N*/A ^1^, 2.17	4.71, 5.47	6.83, 7.07
*Refinement Parameters*				
*R*_1_ (*F*^2^ > 2σ(*F*^2^)), *wR*_2_ (*F*^2^ > 2σ(*F*^2^))	0.0726, 0.1854	0.0810, 0.2135	0.0958, 0.2519	0.1325, 0.3690
*R*_1_ and *wR*_2_ (all data)	0.0905, 0.2027	0.0994, 0.2580	0.1334, 0.2706	0.1757, 0.3923
BASF	–	0.66(1):0.33(1)	–	–
S	1.135	1.207	1.270	1.229
ρ_max_, ρ_min_ (e^−^ Å^−3^)	1.59/−0.95	1.84/−1.30	1.50/−0.60	2.00/−1.48
CSD ^2^	2249854	2249855	2249856	2249857

^1^ The structure was refined using the twin law with the creation of an HKLF5-type reflection file. The MERG code was changed to 0 for compatibility with the HKLF and BASF parameters. ^2^ See also Supplementary Materials.

**Table 2:** Bond distances (Å) in the structural model of Cu oxalates presented in the current study and from the literature data.

Bond, Å	This Study ^1^	Schmittler [26]	Christensen et al. [33]	O’Connor et al. [34]
Cu–O_eq_	1.835(6)–1.856(6)	1.85(8)	1.918(8), 2.081(7)	1.827(3)
Cu–O_ap_	2.512(5)–2.628(5)	2.54(8)	Not reported	2.535(5)
C-O	1.378(13)–1.406(14)	1.37(11)	1.26(1), 1.27(1)	1.314(9)
C–C	1.54(3)–1.58(2)	1.66(8)	1.576(6)	1.705(14)

^1^ A range of values from both structural models is given.

**Table 3:** The main coefficients of the thermal expansion/contraction α_ii_ (*i* = 1–3) × 10^6^ °C^−1^ and orientation of the main axes in the structure of **SM**.

*T* (°C)	*α*_11_	*α*_22_	*α*_33_	<*α*_11_*a*	<*α*_22_*b*	<*α*_33_*c*
30	5.6	47.5	–9.9	31.5	0	172.9
100	19.7	48.9	–4.6	28.6	0	175.7
150	29.8	50.0	–0.8	27.6	0	176.7
200	39.8	51.0	2.9	27.0	0	177.3
240	47.8	51.9	5.9	26.6	0	177.7

Note: *α* is the coefficient of thermal expansion (*α*_11_, *α*_22_ and *α*_33_ are eigenvalues (main values)); <*α*_ii_*a* is the angle between *α*_ii_ and the crystallographic direction [100], calculated with clockwise rotation.

## Data Availability

Not applicable.

## Supplementary Materials

The following supporting information can be downloaded at: https://www.mdpi.com/article/10.3390/ijms24076786/s1, CSD 2249854–2249857: contains the supplementary crystallographic data for this paper.

## References

[1] Baran E.J. (2014). Review: Natural oxalates and their analogous synthetic complexes. J. Coord. Chem..

[2] Frank-Kamenetskaya O.V., Zelenskaya M.S., Izatulina A.R., Vereshagin O.S., Vlasov D.Y., Himelbrant D.E., Pankin D.V. (2021). Copper oxalate formation by lichens and fungi. Sci. Rep..

[3] Frank-Kamenetskaya O.V., Ivanyuk G.Y., Zelenskaya M.S., Izatulina A.R., Kalashnikov A.O., Vlasov D.Y., Polyanskaya E.I. (2019). Calcium Oxalates in Lichens on Surface of Apatite-Nepheline Ore (Kola Peninsula, Russia). Minerals.

[4] Gadd G.M., Bahri-Esfahani J., Li Q., Rhee Y.J., Wei Z., Fomina M., Liang X. (2014). Oxalate production by fungi: Significance in geomycology, biodeterioration and bioremediation. Fungal Biol. Rev..

[5] Chukanov N.V., Pekov I.V. (1996). Moolooite Cu(C_2_O_4_)·H_2_O from the Sarbai deposit—Is the first find in the CIS (The Commonwealth of Independent States). Materials of the Ural Summer Mineralogical School—96.

[6] Syers J.K., Birnie A.C., Mitchell B.B. (1967). The calcium oxalate content of some lichens growing on limestone. Lichenologist.

[7] Clarke R.M., Williams I.R. (1986). Moolooite, a naturally occurring hydrated copper oxalate from Western Australia. Mineral. Mag..

[8] Atencio D., Coutinho J.M.V., Graeser S., Matioli P.A., Menezes Filho L.A.D. (2004). Lindbergite, a new manganese oxalate dihydrate from Boca Rica mine, Galiléia, Minas Gerais, Brazil, and Parsettens, Oberhalbstein, Switzerland. Amer. Mineral..

[9] Vlasov D.Y., Zelenskaya M.S., Izatulina A.R., Janson S.Y., Frank-Kamenetskaya O.V. (2023). Oxalate Crystallization under the Action of Brown Rot Fungi. Crystals.

[10] Purvis O.W. (1984). The occurrence of copper oxalate in lichens growing on copper sulphide-bearing rocks in Scandinavia. Lichenologist.

[11] Chisholm J.E., Jones G.C., Purvis O.W. (1987). Hydrated copper oxalate, moolooite in lichens. Mineral. Mag..

[12] Purvis O.W., Pawlik-Skowronska B., Cressey G., Jones G.C., Kearsley A., Spratt J. (2008). Mineral phases and elemental composition of the copper hyperaccumulator lichen *Lecanora polytropa*. Mineral. Mag..

[13] Vereshchagin O.S., Frank-Kamenetskaya O.V., Vlasov D.Y., Zelenskaya M.S., Rodina O.A., Chernyshova I.A., Himelbrant D.E., Stepanchikova I.S., Britvin S.N. (2023). Microbial biomineralization under extreme conditions: Case study of basaltic rocks, Tolbachik Volcano, Kamchatka, Russia. Catena.

[14] Ren W.-X., Li P.-J., Geng Y., Li X.-J. (2009). Biological leaching of heavy metals from a contaminated soil by *Aspergillus niger*. J. Hazard. Mater..

[15] Glukhova L.B., Frank Y.A., Danilova E.V., Avakyan M.R., Tuovinen O.H., Karnachuk O.V. (2018). Isolation, characterization, and metal response of novel, acid tolerant *Penicillium* spp. from extremely metal-rich waters at a mining site in Transbaikal (Siberia, Russia). Microb. Ecol..

[16] Fomina M., Hillier S., Charnock J.M., Melville K., Alexander I.J., Gadd G.M. (2005). Role of oxalic acid overexcretion in transformations of toxic metal minerals by *Beauveria caledonica*. Appl. Environ. Microbiol..

[17] Tsekova K., Todorova D., Ganeva S. (2010). Removal of heavy metals from industrial wastewater by free and immobilized cells of *Aspergillus niger*. Int. Biodeterior. Biodegr..

[18] Frank-Kamenetskaya O.V., Zelenskaya M.S., Izatulina A.R., Gurzhiy V.V., Rusakov A.V., Vlasov D.Y. (2022). Oxalate formation by *Aspergillus niger* on minerals of manganese ores. Amer. Mineral..

[19] Zelenskaya M.S., Izatulina A.R., Frank-Kamenetskaya O.V., Vlasov D.Y. (2021). Iron Oxalate Humboldtine Crystallization by Fungus *Aspergillus niger*. Crystals.

[20] Donkova B., Mehandjev D. (2005). Thermal-magnetic investigation of the decomposition of copper oxalate-a precursor for catalysts. J. Mater. Sci..

[21] Behnoudnia F., Dehghani H. (2013). Copper(II) oxalate nanospheres and its usage in preparation of Cu(OH)_2_, Cu_2_O and CuO nanostructures: Synthesis and growth mechanism. Polyhedron.

[22] Aimable A., Puentes T., Bowen P. (2011). Synthesis of porous and nanostructured particles of CuO via a copper oxalate route. Powder Technol..

[23] Wu J., Huang K. (2016). Precipitation of flaky moolooite and its thermal decomposition. Int. J. Miner. Metall. Mater..

[24] Singh G., Kapoor I.P.S., Dubey R., Srivastava P. (2012). Preparation, characterization and catalytic effects of copper oxalate nanocrystals. J. Alloys Comp..

[25] Dubicki L., Harris C.M., Kokot E., Martin L. (1966). Magnetic Studies with Copper(II) Salts. VII. The Structure of Copper(II) α,ϖ-Dicarboxylates and Their Amine Derivatives. Inorg. Chem..

[26] Schmittler H. (1968). Zum Strukturprinzip des fehlǵeordneten Kupfer(II)-Oxalats CuC_2_O_4_∙nH_2_O. Monatsber. Dtsch. Akad. Wiss. Berlin.

[27] Fichtner-Schmittler H. (1979). On Some Features of X-Ray Powder Patterns of OD Structures. Krist. Tech..

[28] Michalowicz A., Girerd J.J., Goulon J. (1979). EXAFS Determination of the Copper Oxalate Structure. Relation between Structure and Magnetic Properties. Inorg. Chem..

[29] Fichtner-Schmittler H. (1984). Comments on the Structure of Copper (II) oxalate: Discussion of X-ray Powder Diffraction and EXAFS Results as a Basic for the Interpretation of Magnetic Properties. Cryst. Res. Technol..

[30] Gleizes A., Maury F., Galy J. (1980). Crystal Structure and Magnetism of Sodium Bis(oxalato)cuprate(II) Dihydrate, Na_2_Cu(C_2_O_4_)_2_∙2H_2_O. A Deductive Proposal for the Structure of Copper Oxalate, CuC_2_O_4_∙*x*H_2_O (0 ≤ *x* ≤ 1). Inorg. Chem..

[31] Kondrashev Y.D., Bogdanov V.S., Golubev S.N., Pron G.F. (1985). Crystal Structure of the Ordered Phase of Zinc Oxalate and the Structure of Anhydrous Fe^2+^, Co^2+^, Ni^2+^, Cu^2+^, and Zn^2+^ oxalates. J. Struct. Chem..

[32] Jongen N., Bowen P., Lemaître J., Valmalette J.-C., Hofmann H. (2000). Precipitation of Self-Organized Copper Oxalate Polycrystalline Particles in the Presence of Hydroxypropylmethylcellulose (HPMC): Control of Morphology. J. Colloid Interface Sci..

[33] Christensen A.N., Lebech B., Andersen N.H., Grivel J.-C. (2014). The crystal structure of paramagnetic copper(II) oxalate (CuC_2_O_4_): Formation and thermal decomposition of randomly stacked anisotropic nano-sized crystallites. Dalton Trans..

[34] O’Connor H., Clarke R.M., Kimpton J.A. (2019). Synchrotron radiation diffraction study of the mineral moolooite, and synthetic copper oxalates. Powder Diffr..

[35] (2021). CrysAlisPro Software System.

[36] Jahn H.A., Teller E. (1937). Stability of polyatomic molecules in degenerate electronic states. I. Orbital degeneracy. Proc. R. Soc. A.

[37] Hathaway B.J. (1984). A New Look at the Stereochemistry and Electronic Properties of Complexes of the Copper(II) Ion. Struct. Bond..

[38] Halcrow M.A. (2013). Jahn-Teller distortions in transitional metal compounds, and their importance in functional molecular and inorganic materials. Chem. Soc. Rev..

[39] Burns P.C., Hawthorne F.C. (1996). Static and Dynamic Jahn-Teller effects in Cu^2+^ oxysalts minerals. Can. Mineral..

[40] Kornyakov I.V. (2021). Synthesis and Crystal Chemistry of Novel Mineral-Related Divalent Copper Compound. PhD Thesis.

[41] Dewar M.J.S., Zheng Y.-J. (1990). Structure of the Oxalate Ion. J. Mol. Struct. THEOCHEM.

[42] Naumov D.Y., Podberezskaya N.V., Boldyreva E.V., Virovets A.V. (1996). Crystal-Chemical Analysis of the Structures of Oxalic Acid and its Salts M*_x_*(C_2_O_4_)*_y_*∙*n*H_2_O (*n* = 0-3). J. Struct. Chem..

[43] Izatulina A.R., Gurzhiy V.V., Krzhizhanovskaya M.G., Kuz’mina M.A., Leoni M., Frank-Kamenetskaya O.V. (2018). Hydrated Calcium Oxalates: Crystal Structures, Thermal Stability, and Phase Evolution. Cryst. Growth Des..

[44] Proffen T., Neder R.B. (1997). *DISCUS*: A program for diffuse scattering and defect-structure simulation. J. Appl. Cryst..

[45] Neder R.B., Proffen T. (2007). Diffuse Scattering and Defect Structure Simulations—A Cook Book Using the Programs DISCUS.

[46] Diffuse Program Collection. https://github.com/tproffen/DiffuseCode.

[47] Welberry T.R., Butler B.D. (1994). Interpretation of Diffuse X-ray Scattering via Models of Disorder. J. Appl. Cryst..

[48] Welberry T.R. (2004). Diffuse Scattering and Models of Disorder.

[49] Hahn T. (2002). The 230 space groups. International Tables for Crystallography Volume A: Space-Group Symmetry.

[50] Calos N.J., Forrester J.S., Schaffer G.B. (1996). A Crystallographic Contribution to the Mechanism of a Mechanically Induced Solid State Reaction. J. Solid State Chem..

[51] Schmittler H. (1969). Personal communication.

[52] Dollimore D. (1987). The Thermal Decomposition of Oxalates. A Review. Thermochim. Acta.

[53] Higashiyama T., Hasegawa S. (1971). The Differential Thermal Analysis of Potassium Oxalate. Bull. Chem. Soc. Jpn..

[54] Davies A.K., Gilligan J.V., Jones S.A. (1996). Characterisation And Thermal Analysis Of Vanadium(II) Oxalate. J. Therm. Anal..

[55] Frost R.L., Weier M.L. (2003). Thermal treatment of weddellite—A Raman and infrared emission spectroscopic study. Thermochim. Acta.

[56] Frost R.L., Weier M.L. (2004). Thermal treatment of whewellite—A thermal analysis and Raman spectroscopic study. Thermochim. Acta.

[57] Frost R.L., Erickson K., Weier M.L. (2004). Thermal treatment of moolooite. A high resolution thermogravimetric and hot stage Raman spectroscopic study. J. Therm. Anal. Calorim..

[58] Glasser L. (2019). Effective Volumes of Waters of Crystallization: Ionic Systems. Cryst. Growth Des..

[59] Fraser W. (2020). Diffractometers for modern X-ray crystallography: The XtaLAB Synergy X-ray diffractometer platform. Rigaku J..

[60] Sheldrick G.M. (2015). SHELXT—Integrated space-group and crystal structure determination. Acta Crystallogr..

[61] Sheldrick G.M. (2015). Crystal structure refinement with SHELXL. Acta Crystallogr..

[62] Dolomanov O.V., Bourhis L.J., Gildea R.J., Howard J.A.K., Puschmann H. (2009). OLEX2: A complete structure solution, refinement and analysis program. J. Appl. Cryst..

[63] Turner M.J., McKinnon J.J., Jayatilaka D., Spackman M.A. (2011). Visualisation and characterisation of voids in crystalline materials. CrystEngComm.

[64] Spackman P.R., Turner M.J., McKinnon J.J., Wolff S.K., Grimwood D.J., Jayatilaka D., Spackman M.A. (2021). *CrystalExplorer*: A program for Hirshfeld surface analysis, visualization and quantitative analysis of molecular crystals. J. Appl. Cryst..

[65] Topas 5.0 (2014). General Profile and Structure Analysis Software for Powder Diffraction Data.

[66] Langreiter T., Kahlenberg V. (2015). TEV—A Program for the Determination of the Thermal Expansion Tensor from Diffraction Data. Crystals.

